# ALK expressed in a gastrointestinal stromal tumor harboring PDGFRA p. D842V mutation:a case report

**DOI:** 10.1186/s13000-020-0926-x

**Published:** 2020-01-31

**Authors:** Jun Fan, Ming Yang, Bo Huang, Zhenkao Wang, Danju Luo, Jiwei Zhang, Peng Zhang, Heshui Shi, Yan Li, Xiu Nie

**Affiliations:** 1 Department of Pathology, Union Hospital, Tongji Medical College, Huazhong University of Science and Technology, 1277 Jiefang Avenue, Wuhan, Hubei China; 2 Department of Gastrointestinal surgery, Union Hospital, Tongji Medical College, Huazhong University of Science and Technology, Wuhan, 430022 Hubei China; 3 Department of Radiology, Union Hospital, Tongji Medical College, Huazhong University of Science and Technology, Wuhan, 430022 Hubei China

**Keywords:** Gastrointestinal stromal tumors, *PDGFRA* D842V mutation, ALK expression

## Abstract

**Background:**

Gastrointestinal stromal tumors (GISTs) are the most common type of adult mesenchymal neoplasms. The events that drive GIST oncogenesis are primarily *KIT* or *PDGFRA* mutations, which lead to the susceptibility of these tumors to small-molecule tyrosine kinase inhibitors such as imatinib and sunitinib. However, previous studies have shown that patients with a *PDGFRA* D842V mutation in GISTs have a very low rate of response to imatinib treatment. Therefore, novel tyrosine kinase inhibitors (TKIs) are currently being evaluated in clinical trials to treat GISTs harboring a *PDGFRA* D842V mutation. Anaplastic lymphoma kinase (ALK) overexpression was not expected to be present in the GIST, and it has been used as a biomarker to distinguish GISTs from other types of mesenchymal tumors.

**Case presentation:**

Here, we report a 37-year-old male patient who presented with a large mass in the right upper abdomen and was subsequently diagnosed with a GIST harboring a *PDGFRA* D842V mutation. We unexpectedly found that the GIST in this patient exhibited simultaneous ALK expression.

**Conclusions:**

This is the first case reported of a GIST with ALK expression. This rare phenomenon suggests that the diagnosis of a GIST cannot be excluded absolutely if a tumor exhibits ALK expression. In addition, ALK may be a potential therapeutic target for patients with imatinib-resistant stromal tumors.

## Background

A gastrointestinal stromal tumor (GIST) is a type of mesenchymal tumor that arises throughout the gastrointestinal tract [1]. Up to 80% of GISTs carry pathogenic activating mutations of the proto-Oncogene c-Kit (*KIT*), while 5 to 10% harbor activating mutations of platelet-derived growth factor receptor alpha (*PDGFRA*); both are members of the type III tyrosine kinase receptor family [2, 3].

The use of KIT/PDGFRA tyrosine kinase inhibitors (TKIs) has transformed the therapeutic pattern of localized and advanced GIST. In general, tumors with *KIT* exon 11 mutations are most sensitive to imatinib, whereas GISTs harboring a mutation in *PDGFRA* exon 18 (p.D842V) are considered imatinib-resistant [4, 5]. Molecular modeling of *PDGFRA* D842V suggests that the mutant protein binds imatinib with a lower affinity than the wild-type structure [6, 7]. To our knowledge, GISTs harboring *PDGFRA* D842V do not possess any actionable recurrent molecular events of therapeutic significance. Therefore, it is necessary to explore new therapeutic targets for patients with drug-resistant GIST harboring *PDGFRA* D842V.

Anaplastic lymphoma kinase (ALK), belonging to the insulin receptor superfamily, is a transmembrane receptor tyrosine kinase. Overexpression of ALK, which is associated with oncogenesis, can be caused by gene fusion, mutations and amplification. The rearrangements of the ALK gene have been implicated in the pathogenicity of a number of neoplasms that include anaplastic large cell lymphoma (ALCL), a subset of pulmonary adenocarcinoma, inflammatory myofibroblastic tumor (IMT), and epithelioid fibrous histiocytoma (EFH); the rearrangements result in fusion proteins that constitutively activate the ALK tyrosine kinase domain [8–10]. In particular, approximately 50% of IMTs are correlated with *ALK* rearrangements [11]. Several studies have indicated that targeting ALK with kinase inhibitors, such as crizotinib/ceritinib, is a potential treatment option [12, 13]. However, few studies have reported the expression of ALK in patients with drug-resistant GIST harboring *PDGFRA* D842V. Previously, it was reported that ALK was not found in GISTs, and ALK staining was applied as a way to distinguish GIST from IMT [14]. In the present report, one case was described of a 37-year-old man with GIST harboring the D842V mutant, in which ALK was expressed.

## Case presentation

**Fig. 1:**
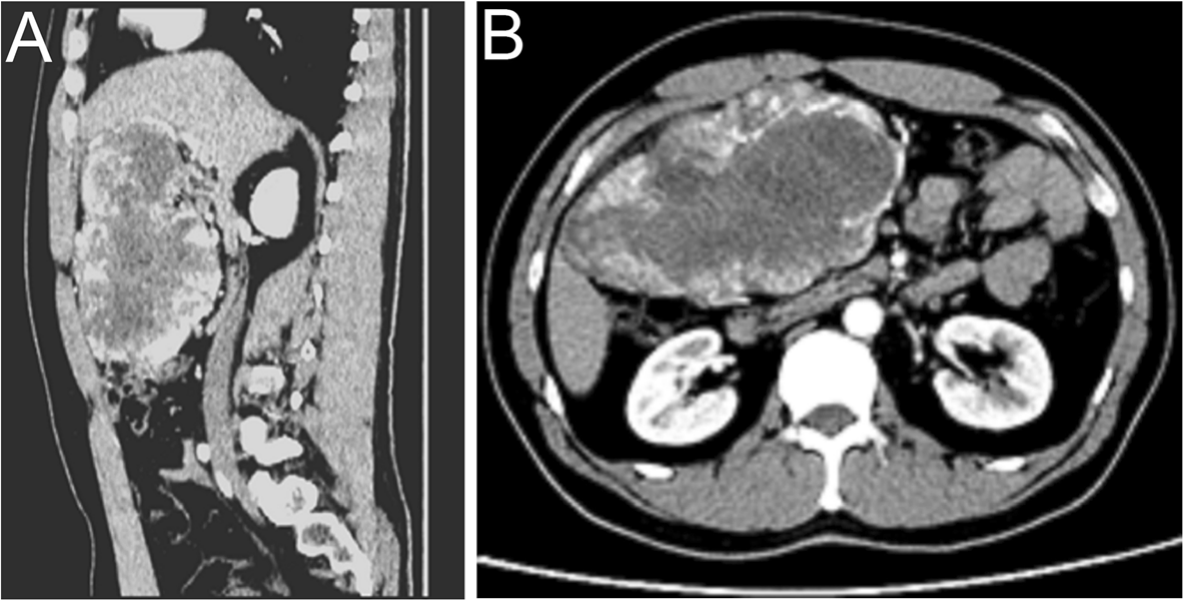
Abdominal CT scan shows a mass located in the right upper abdominal cavity and its three-dimensional reconstruction image. **a**: sagittal position, **b**: transverse position

A 37-year-old male patient presenting with abdominal distention for more than 10 days without abdominal pain, diarrhea, nausea or vomiting was admitted to our hospital. Computed tomography (CT) showed a large irregular mass located in the right upper abdominal cavity (Fig. 1). The mass was uneven in density, with CT values ranging from 20 to 45 HU. Its edges were nodular exogenous protrusions with an estimated size of 16.2 × 15.4 × 8.8 cm. After contrast infusion, the edges of the lesion and the gastric antrum were found to be blurred and exaggerated. Many blood vessels wrapped around the juncture. There was no sign of invasion to the right lobe or caudate lobe of the liver, gallbladder, duodenum or head and neck of the pancreas on enhanced scan, and no thickening of the adjacent peritoneum was observed.

**Fig. 2:**
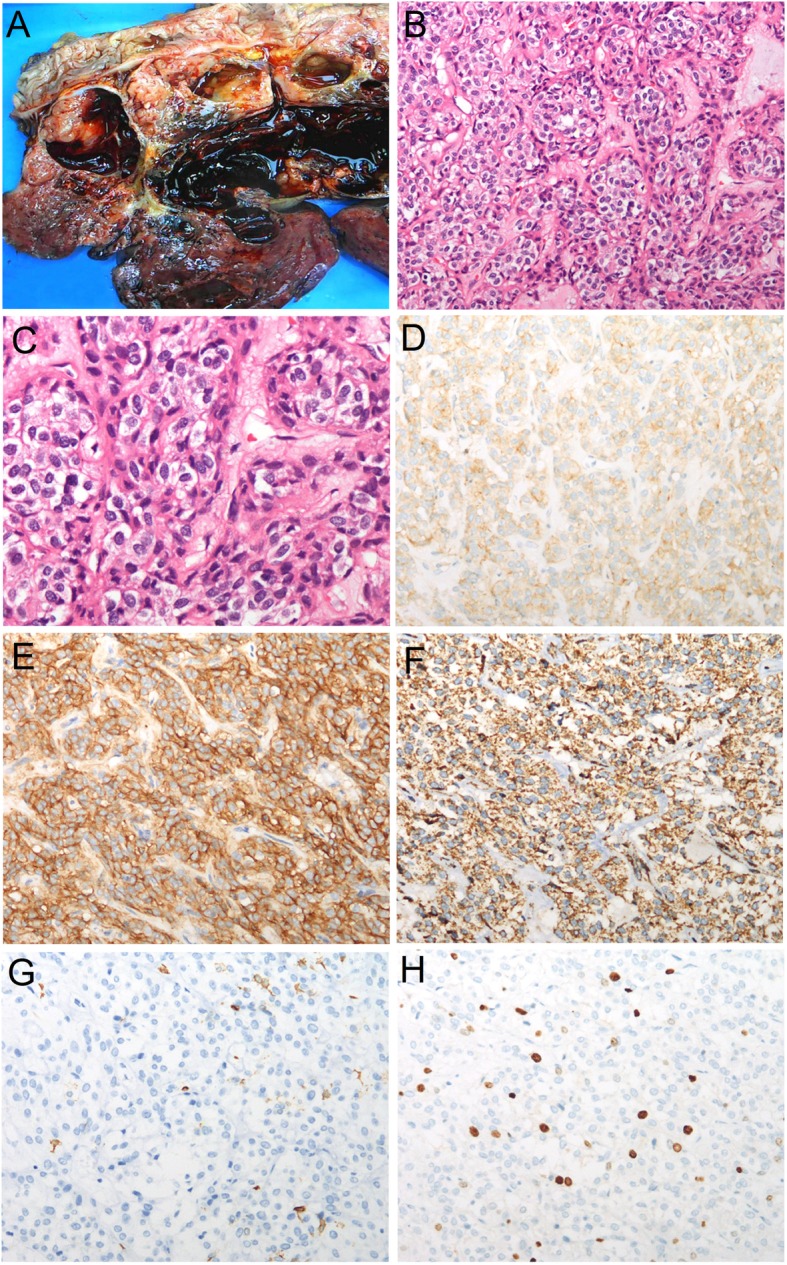
Gross and microscopic images of the GIST. This tumor possessed both cystic and solid components. The cystic cavity was filled with dark brown blood, and the solid areas were delicate in texture (**a**). Sections of the mass were observed under a microscope, and the tissue was characterized by epithelioid tumor cells that were arranged in a prominent nesting pattern (**b**, H&E stain, 100×). Images of epithelioid tumor cells stained by H&E under a high-power field are displayed in **c** (200×). The tumor cells exhibited a positive cytoplasmic signal for CD117 (**d**, IHC, 200×), DOG-1 (**e**, IHC, 200×) and SDHB (**f**, IHC, 200×), and they were negative for SMA (**g**, IHC, 200×). The Ki-67 labeling index was estimated to be 10% (**h**, IHC, 200×)

**Fig. 3:**
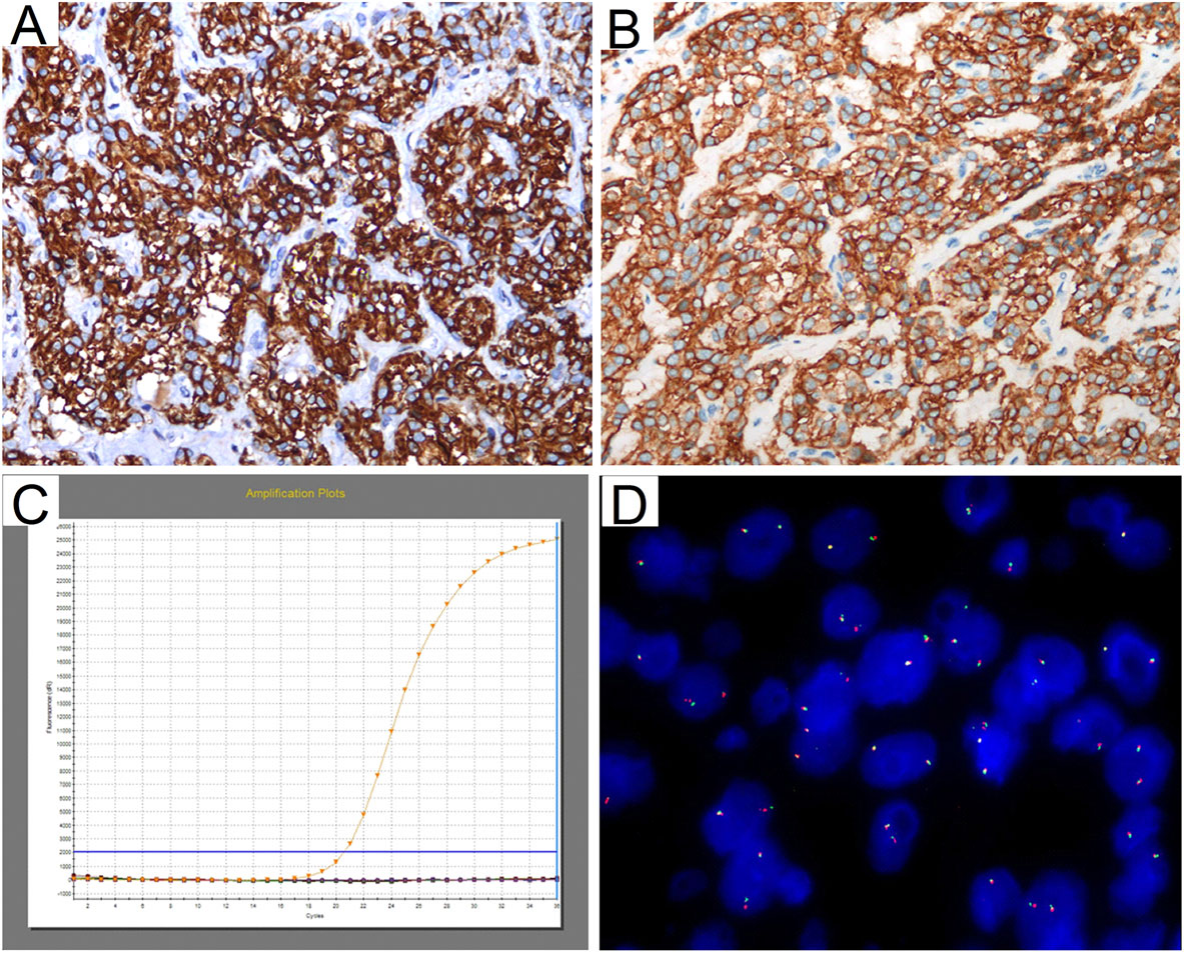
ALK expression and the *ALK* genotype in GIST of the current case. Immunohistochemical staining with ALK antibodies demonstrated diffuse ALK expression within epithelioid tumor cells. Staining results for the D5F3 clone (**a**, IHC, 200×) and 1A4 clone (**b**, IHC, 200×) are displayed. No fusion of *ALK* was detected by ARMS-PCR (**c**). A break-apart fluorescent in situ hybridization (FISH) assay did not find *ALK* rearrangement (**d**, FISH, 1000×)

**Fig. 4:**
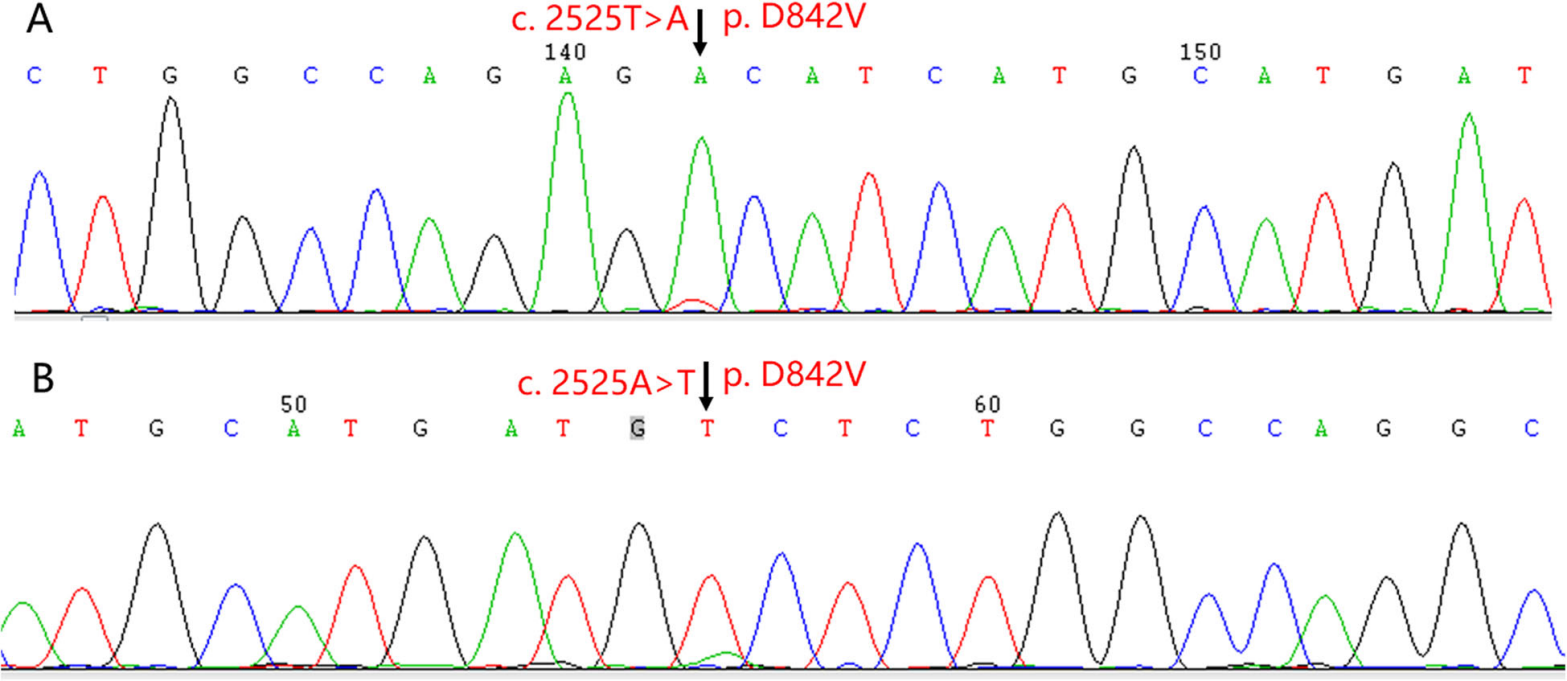
Sanger sequencing detected a c.2525A > T, p.D842V mutation at exon 18 of *PDGFRA* for the GIST in this case. Panel **a** displays the forward sequencing result, and Panel **b** reveals the reverse sequencing result

A laparotomy was performed to remove the tumor and part of the colon that was involved. Upon gross examination, the removed mass, which was located in the mesentery of the colon, was found to be approximately 18 × 17 × 8 cm in size, and its capsule was intact (Fig. 2a). A cross section analysis revealed multilocular cyst formation, bleeding, and necrosis in some areas. However, the lesion remained solid in some other areas; it was delicate in texture and had papillae protruding from the inner wall. Histologically, epithelioid tumor cells were arranged in a prominent nesting pattern (Fig. 2b-c), and they showed signs of regional cystic degeneration, hemorrhage and necrosis. Tumor cells were positive for CD117 (weakly positive, Fig. 2d), DOG-1 (Fig. 2d) and SDHB (Fig. 2f), but they were negative for pancytokeratin, CD34, SMA (Fig. 2g), S-100 and Calretinin. Ki-67 labeling was estimated to be 10% (Fig. 2h), and the mitotic count was performed in an area of more than 5/5 mm^2^. These findings, especially the presence of DOG-1 and CD117, supported the diagnosis of GIST originating from the mesentery of the colon, and its recurrence risk was classified as high [15]. To exclude epithelioid IMT, two different antibody clones, D5F3 and 1A4, were used (Fig. 3a and b) to detect ALK expression. In contrast to our expectations, ALK was strongly stained by both antibodies. However, no gene fusion or copy number increase was detected in *ALK* by amplification refractory mutation system (ARMS) PCR (Fig. 3c), fluorescent in situ hybridization (FISH) (Fig. 3d) and next-generation sequencing technology. Sanger sequencing revealed the presence of the p.D842V mutation in exon 18 of *PDGFRA* (Fig. 4). PDGFRA-driven, p.D842V-mutated GIST has been found to react poorly to small-molecule tyrosine kinase inhibitors such as imatinib. Moreover, drugs directed against PDGFRA D842V mutations have not been approved clinically. Therefore, follow-up was suggested for this patient. However, 6 months after surgery, the patient had a relapse.

## Discussion

Here, we present a case of a giant tumor that occurred in the greater omentum. Pathological examination revealed that the tumor mainly consisted of round or oval epithelioid cells. Based on the location of the lesion and the morphology of the tumor cells, the diagnosis of epithelioid GIST was first taken into consideration; epithelioid GIST is the most common category of mesenchymal tumors of the gastrointestinal tract. Second, we excluded the diagnosis of leiomyoma and mesothelioma based on the results of immunohistochemical staining (IHC). IHC results showed that the two specific markers for GIST, CD117 and DOG1, were positive, while SMA and other indicators were negative, all of which supported the diagnosis of epithelioid GIST. Sanger sequencing results demonstrated that the lesion harbored a PDGFRA mutation in exon 18 (p.D842V). This further confirmed the diagnosis of GIST. There was a lack of inflammatory cell infiltration around tumor cells, which was useful in ruling out inflammatory myofibroblastic tumors with epithelial morphology. ALK overexpression is detected in 50–60% of IMT cases [16]. Therefore, ALK positivity is helpful in the diagnosis of IMT. To our surprise, the tumor in the current report exhibited strong and diffuse staining for ALK that was confirmed by antibodies from two different clones. In general, immunostaining allows differentiation of IMT and GIST. Most IMTs are positive for ALK and negative for CD117/DOG1, whereas most GISTs are ALK-negative and CD117/DOG1-positive. The tumor cells in our case were positive for both ALK and CD117, and it was thus difficult to determine whether the tumor was an IMT or a GIST. To the best of our knowledge, only one previous study examined the ALK status of GISTs and found that this type of tumor was ALK-negative [17]. Therefore, this was the first time an ALK-positive GIST was reported. Next, we tried to determine whether the mechanism underlying the expression of ALK in this GIST case was similar to that of inflammatory myofibroblastic tumors, which are mainly caused by the fusion of *ALK*. Unexpectedly, the FISH results were negative after two tests, with only a small fraction of positive isolated signals in tumor cells and no gain of *ALK* copy number. We eventually diagnosed the tumor as a GIST because the PDGFRA mutation was confirmed by Sanger sequencing and CD117/DOG1 were positive in the IHC test. In addition, ALK gene rearrangement and SMA expression were negative, which further confirmed that our case was a GIST. For further confirmation, NGS was performed, and neither *ALK* gene fusion nor copy number gains were detected. Based on the results of both FISH and NGS, it was concluded that ALK expression in this GIST case was not caused by gene fusion or amplification. The molecular basis for ALK immunoreactivity in our case is not known. Alison and colleagues reported that ALK expression in angiomatoid fibrous histiocytoma (AFH) was common (9/11 by antibody clone number D5F3), but none of their cases with positive IHC staining of ALK showed evidence of *ALK* rearrangement or copy number gain by FISH [18]. The discrepancy of DNA and protein abnormalities remains to be explored. A recent report identified that copy-number variations (CNVs) of *PDGFRA* might be implicated in the resistance of ALK-positive non-small cell lung cancer to targeted therapies [19]. Another study showed that PDGFR inhibition was a rational and effective therapeutic agent for nucleophosmin-anaplastic lymphoma kinase (NPM-ALK) fusion lymphomas, suggesting that ALK might be a target of activating mutations of the genes encoding PDGFRA [20]. The correlations of ALK and PDGFRA in tumorigenesis and therapeutic potential need to be clarified in the future.

ALK inhibitors have shown remarkable effects against ALK-driven tumors. Several studies have shown that crizotinib exhibits impressive therapeutic effects in patients with *ALK*-translocated IMT [11, 21]. These results support the dependence of *ALK*-rearranged tumors on ALK-mediated signaling and suggest a therapeutic option for genetically identified patients with the aggressive form of this soft-tissue tumor. In addition, Noah A. Cohen found that imatinib increased the expression of activated MET in imatinib-sensitive human GIST cell lines and in a genetically engineered mouse model of GIST. Moreover, the combination of crizotinib and imatinib was more effective than imatinib alone in imatinib-sensitive GIST models. Finally, cabozantinib, a dual MET and KIT small-molecule inhibitor, was remarkably more effective than imatinib when used in multiple preclinical models of imatinib-sensitive and imatinib-resistant GISTs [22]. Although ALK was substantially expressed in this case, no gene rearrangement or amplification was detected. An increasing number of studies have shown that ALK IHC-positive and FISH-negative tumors may still respond to crizotinib [13, 23]. Together, these data suggest that ALK inhibitors may be a potential therapeutic option for ALK-positive p.D842V mutation-harboring GIST. In addition, patients with *PDGFRA* mutations, including mutations resulting in the substitution alteration at Asp842, had a lower risk of recurrence than patients with *KIT* mutations [24]. In this case, however, the tumor recurred 6 months after the operation, indicating that overexpression of *ALK* may play an important role in the early recurrence of GIST harboring *PDGFRA* mutations.

In summary, we reported the first case of GIST with ALK expression, which may present as a potential source of diagnostic confusion, as it could be mistaken for IMT. The mechanism underlying ALK expression is still unknown, with ALK rearrangement or amplification being excluded. Further studies are warranted to elucidate the mechanism and significance of ALK expression in these tumors.

## Data Availability

The dataset supporting the conclusions of this article is included within the article.

## Abbreviations

ALK: Anaplastic lymphoma kinase
CT: Computed tomography
GIST: Gastrointestinal stromal tumors
H&E: Hematoxylin-Eosin
IHC: Immunohistochemical staining
PDGFRA: Platelet-derived growth factor receptor alpha
